# Optimization and prioritization of paediatric drugs for visceral leishmaniasis

**DOI:** 10.3389/fped.2025.1635252

**Published:** 2025-09-04

**Authors:** Tiziana Masini, Ana Nilce Silveira Maia-Elkhoury, Dinesh Mondal, Piero Olliaro, Khechar N. Paudel, Martina Penazzato, Samantha Yuri Oshiro Branco Valadas, Aya Yajima, Abate Beshah, Supriya Warusavithana, Saurabh Jain

**Affiliations:** ^1^ World Health Organization, Research for Health Department, Science Division, Geneva, Switzerland; ^2^ Pan American Health Organization - World Health Organization, Regional Office of the Americas, Duque de Caxias- Rio de Janeiro, Brazil; ^3^ icddr,b, 68 Shaheed Tajuddin Ahmed Saranai, Mokakhali, Dhaka, Bangladesh; ^4^ International Severe Acute Respiratory and emerging Infection Consortium, Pandemic Sciences Institute, University of Oxford, Oxford, United Kingdom; ^5^ Province Hospital, Surkhet, Nepal; ^6^ World Health Organization, Regional Office for South-East Asia, New Delhi, India; ^7^ World Health Organization, Regional Office for Africa, Brazzaville, Republic of Congo; ^8^ World Health Organization, Regional Office of the Eastern Mediterranean, Cairo, Egypt; ^9^ World Health Organization, Malaria and Neglected Tropical Disease Department, Geneva, Switzerland

**Keywords:** visceral leishmaniasis, Kala-azar, paediatrics, miltefosine, amphotericin B

## Abstract

Visceral leishmaniasis (VL) is a fatal disease if left untreated. Globally, at least 50% of VL cases are reported to be children younger than 15 years, with a higher incidence among males. VL is intrinsically associated with poverty and poor social determinants of health. Malnutrition and immune suppression are risk factors for severe VL, making children particularly vulnerable to this disease. Available treatment options vary depending on the eco-epidemiological context, but are in general suboptimal, especially for children. In 2023, the World Health Organization convened a paediatric drug optimization exercise (PADO) for VL, bringing together more than 60 experts globally in the field of VL to identify formulations of VL medicines to prioritize for development to address the specific needs of children. The group prioritized a 20 mg scored, dispersible tablet formulation of miltefosine and an oral solid dosage form of amphotericin B, acknowledging recent developments in allometric dosing for miltefosine and ongoing research for the development of oral amphotericin B. For miltefosine, this prompted an ongoing update of the WHO Prequalification Expression of interest to promote generic manufacturing. A compound with a new mechanism of action, LXE408, which is currently being investigated in Phase II, was included in the PADO watch list, signalling that paediatric investigations should start as soon as enough data are available from adult studies, not to delay access to latest available innovations for children with VL.

## Introduction

Visceral leishmaniasis (VL), also known as Kala-azar, is a poverty-related parasitic vector-borne disease that is endemic in 80 countries in the world with an estimated 50,000–90,000 new cases per year (1). Its chronic nature, outbreak and high-mortality potential make it disproportionately endemic in low- and lower-middle-income countries, afflicting their poorest and most isolated populations. The disease is associated with malnutrition, population displacement, migration of non-immune people to endemic areas, poor housing, illiteracy, a weakened immune system, and lack of resources (2–4).

VL causes high disease burden in the three major eco-epidemiological hotspots – the Indian subcontinent, Brazil and Eastern Africa, which accounted for 6%, 14% and 74% of the global burden in 2023, respectively. With the success of the Kala-azar elimination programme in the Indian subcontinent, the main burden of VL has now shifted to Eastern Africa (5).

Treatment of VL depends on several factors, including the type of patients (new, relapse), the type of *Leishmania parasite species*, associated comorbid conditions, a person's immune status (e.g., people living with HIV) and other immune suppression conditions (1). As a result, treatment options vary across geographical regions (Supplementary Table S1).

Children under 15 years of age constitute approximately 50% of all VL cases. Malnutrition and immune suppression predispose to clinical disease, especially among children who are young and malnourished and act as a risk factor for severe VL (2, 4).

Except for miltefosine which is an oral drug, other antileishmanial drugs are given parenterally and require hospitalization or a visit to receive the injection, and the dosing is weight-based. Most VL drugs are available from a single supplier and are not registered in several endemic countries, with severe implications on costs and availability. Only liposomal amphotericin B (LAmB) is supplied through a donation programme in a few countries, while there is no donation of other antileishmanial drugs. Cold chain maintenance, storage and supply chain are also an issue for some of these medicines.

Currently available treatment options are particularly not suitable for children, which prompted the need to discuss how to focus limited resources on the most needed formulations for a population that is so severely affected by VL.

## Paediatric Drug Optimization (PADO) for visceral leishmaniasis

In June 2023, WHO convened a PAediatric Drug Optimization (PADO) (6) meeting for VL with 60 experts from five WHO regions, including researchers, funders, implementing partners, national programme managers, regulators and product development partnerships to review drugs and formulations currently used for VL and evaluate their appropriateness for children, review ongoing studies and efforts to develop paediatric formulations as well as VL drugs currently under investigation (7).

Meetings focusing on optimization of paediatric medicines have been successfully convened by WHO in several disease areas including HIV, tuberculosis, hepatitis C, COVID-19 and antibiotics, showing the impact that such a consensus-building process can have on focusing research and development efforts and resources.

The aim of the PADO-VL meeting was to reach a consensus on priority formulations of medicines for VL to be investigated and developed with a time horizon of 3–5 years (PADO priority list), identify promising candidates for investigation and development for children with a time horizon of 5–10 years (PADO watch list), as well as agree on a clear research agenda to support and enable future optimization work.

The PADO for VL meeting was part of a broader PADO exercise for neglected tropical diseases (NTDs) which focused on NTDs with the highest burden in the paediatric population, including schistosomiasis, human African trypanosomiasis, scabies and onchocerciasis (7).

The PADO-VL group reviewed each of the available antileishmanial drugs for their efficacy and use across different epidemiological regions, their available formulations, toxicity profiles and their suitability for children. The group noted that therapeutic efficacy study designs and their results in VL are complex with substantial variations in terms of inclusion and exclusion criteria, definitions for disease diagnosis and treatment outcomes. The drug trials mostly use linear dosing in children–therefore, generating potentially inadequate regimens. Adapted dosing regimens and formulations are generally lacking for children.

Except for miltefosine, antileishmanial drugs are administered parenterally: intramuscular injections are painful and often unsuited, given that many children with VL are severely malnourished and have little or no muscle mass, with a risk of muscle abscess and nerve damage; venous access for intravenous infusion is equally challenging – hence the risk of thrombophlebitis of vein. For miltefosine, acceptability in younger children who cannot swallow capsules is also poor, and capsules need protection from moisture, which is not ideal for use in tropical climates. In addition, oral miltefosine cannot be prescribed to women of childbearing age if pregnancy status cannot be verified and adequate contraception cannot be instituted during treatment and five months after the last dose, as the drug has a potential teratogenic effect – thus further limiting its use in adolescent girls. Gastrointestinal effects including vomiting and/or diarrhoea are common during miltefosine administration and may result in volume depletion (8).

Other combinations of available drugs have been studied in Africa and in Brazil, including in children, but target efficacy was not achieved for any of these regimens (9–11).

## Discussion

### PADO priority list for visceral leishmaniasis

To overcome low exposure and reduced efficacy in younger children at the conventional 2.5 mg/kg/day dose, recent trials investigated miltefosine given at allometric doses ranging from a 20 mg to 80 mg daily dose (divided into two daily administrations to reduce vomiting). This allometric dose regimen has shown high efficacy in children and in malnourished individuals with VL in Eastern Africa demonstrating that it leads to an equivalent exposure to the one observed in adults and it was reviewed in the context of a recent guideline development group convened by WHO (12–15). Considering future potential recommendations for allometric dosing of miltefosine, as well as the low acceptability and dose flexibility of currently available miltefosine formulations (i.e., 50 mg and 10 mg capsules), an age-appropriate formulation of oral miltefosine – 20 mg scored, dispersible tablets – was identified as a priority formulation to develop for children with visceral leishmaniasis in the short term (3–5 years) (Table 1). Dispersible tablets were preferred over other dosage forms such as oral liquids or syrups, after reflecting on well-known issues with such dosage forms related to procurement and supply, as well as dosing and stability (16). This formulation would also allow for doses in multiples of 20 mg and 10 mg, which would facilitate allometric dosing of miltefosine.

**Table 1:** Paediatric drug optimization (PADO) priority list, watch list and research priorities for visceral leishmaniasis.

Paediatric drug optimization for visceral leishmaniasis	
Priority list	
*Priority formulations to be investigated and developed for children within a time horizon of 3–5 years (short-term)*	
**Miltefosine** 20 mg scored, dispersible tablets	**Amphotericin B** Oral dosage form (details to be decided based on ongoing studies)
Watch list	
*Priority formulations to be investigated and developed for children within a time horizon of 5–10 years (long-term)*	
LXE408	
Research priorities for visceral leishmaniasis	
• investigating the effect of the COVID-19 pandemic as well as other epidemics, natural disasters and social and political disruptions on the VL burden for children. • investigating parasite resistance for amphotericin B and any implications for the clinical management of people with VL, including children. • extending studies with allometric doses of miltefosine for people with VL that enable pragmatic dosing strategies while achieving target exposure. • developing a simplified weight band–based dosing table for allometric miltefosine for children, including the evaluation of harmonized weight bands used for other diseases. • investigating the development of miltefosine dosage forms with improved gastrointestinal tolerability. • exploring the value of using anti-inflammatory drugs as co-adjuvant treatment for VL. • investigating the effect of increased doses of LAmB in children with VL relapses; and • establishing exposure–response (PK and pharmacodynamic) relationships in VL in relation to the target exposure for children, with studies being undertaken in all regions where VL is endemic to consider regional specificities and their potential effects on medicine PK.	

The group acknowledged that miltefosine is not currently recommended for VL in the region of the Americas as trials showed 42% cure rate at 28 days of treatment and 68% at 42 days of treatment (17, 18). Moreover, reliable data on the efficacy of miltefosine in VL in the Mediterranean region are lacking. Nevertheless, a paediatric formulation of miltefosine was still considered a priority for further development, as it remains the only available oral treatment option for VL, which can be utilized in most endemic settings.

The addition of this formulation of miltefosine to the PADO priority list prompted an ongoing update of the WHO Prequalification expression of interest to promote generic manufacturing of this medicine. Investigating the development of miltefosine dosage forms with reduced gastrointestinal side effects was noted as a key priority.

An oral formulation of amphotericin B that is cost-effective, safe, stable at tropical temperatures, accessible, and easy to administer, was also prioritized for development in the short term, with the characteristics – dosage form, strength – of this formulation pending upon results of ongoing studies (Table 1). In particular, a Phase II study exploring amphotericin B loaded in cochleates – to delay release in gastrointestinal media – for cryptococcal meningitis (19, 20) and Phase Ia, Ib studies to evaluate the safety, tolerability and PK of novel lipid-based self-emulsifying oral amphotericin B formulations including syrup and capsule formulations that have already proved stable at tropical temperatures (21–23). More data are needed to understand exposures with new oral formulations of amphotericin B to ensure that exposure is optimized to achieve optimal outcomes at non-toxic dosing regimens.

Potential co-formulations of VL medicines were also discussed as part of the PADO for VL meeting. However, the group agreed that standalone formulations of medicines for VL allow for greater flexibility in the combinations to use, which is particularly important considering that different treatment options are used in different geographic settings.

### PADO watch list for visceral leishmaniasis

New treatments that are currently being studied for VL aim to move away from existing drugs to new effective, oral, safe and easy-to-use treatments. In particular, these compounds are being studied as oral, well-tolerated and safe treatments with improved efficacy that can be used at the primary health care centre level and are affordable. Such new treatments can benefit all clinical forms of leishmaniasis and also benefit people with PKDL and VL-HIV co-infected patients.

While most new chemical entities are still in Phase I, compound LXE408 is in a more advanced stage of development (Phase II). LXE408 is a kinetoplastid-selective proteasome inhibitor that has shown good tolerability with no specific safety concerns for its investigation in children. The paediatric formulation that is being explored is a minitablet. Considering this, LXE408 was included in the PADO watch list for VL, signalling that once enough data are available from adult studies to initiate paediatric investigations, this should be done promptly to ensure that children with VL benefit from the latest available innovations (Table 1).

As most compounds for VL are undergoing early clinical development, a preliminary conversation on preferred product characteristics was initiated during the meeting (Table 2). The group noted that the lowest age for a VL therapeutic indication should be 2 years of age, even though expanding the study population to children down to 6 months of age would be relevant, especially for settings where a substantial proportion of people in need for VL treatment are children less than 2 years of age such as Sudan and countries in other endemic geographies. It was also acknowledged that obtaining ethical approvals and including this age group in clinical trials – whether for VL or other NTDs – presents significant challenges (24). There was a general consensus that drug formulations to treat VL in children should be oral formulations that are acceptable for children across the paediatric age spectrum, and stable at room temperature – eliminating the need for refrigeration to facilitate procurement and streamline logistics at country level. Indeed, there is an expectation that cost-effective, orally administered formulations that are stable at room temperature will significantly facilitate access and simplify distribution. Furthermore, the introduction of such formulations should be accompanied by efforts to build the capacity of healthcare personnel and to strengthen pharmacovigilance systems.

**Table 2:** First draft of proposed preferred product characteristics for medicines used in children with VL.

Attribute	Minimum target	Optimal target
**Lowest age for the VL therapeutic indication**	Two years	Six months
**Dosage form**	Oral or intramuscular	Oral
**Toxicity**	No severe adverse events, no gastrointestinal side effects, low renal and hepatic toxicity and no food intake necessary	No side effects
**Frequency of administration**	Twice daily	Once daily
**Drug-drug interactions (DDIs)**	No DDi with antibiotics, TB drugs, ARVs and drugs of common use	No DDi
**Laboratory monitoring requirement**	Basic diagnostic available at point of care (haematological, kidney function test, liver function test, blood sugar, ECG)	No laboratory monitoring required
**Efficacy**	Non-inferior to standard of care (approx. 90%)	Superior to existing standard of care (approx. 95%)
**Geographical coverage**	VL-endemic areas Against one of the Leishmania species (*L. infantum* or *L. donovani*)	All areas of VL outbreaks Active against *L. infantum* and *L. donovani* (full geographical coverage)
**Cost for high-burden countries**	Similar to existing treatments	Similar or cheaper than existing treatments and no management cost for patients
**Stability and shelf life**	Room temperature, 1–2 years of shelf life	No cold chain required, storage up to 40 °C and shelf life of 3–5 years
**Barrier to resistance (defined as the risk of reduced or lost drug efficacy against previously susceptible Leishmania due to molecular changes, even at the highest tolerated doses)**	*Various opinions:* Low/moderate *in vivo* resistance risk documented High Less than 10% Active against one resistant strain	Should be active against any resistant strains and should not induce any resistance; no cross resistance
**Other characteristics**		Use of polymeric and metallic nanocarrier-based strategies such as macrophage targeting, organ targeting, enhanced oral bioavailability and photodynamic therapy No contraindication for pregnant and lactating women, no need for contraception in women of child-bearing potential

### Research priorities

Following the priority-setting exercise, the PADO-VL group discussed research gaps for VL medicines for children to address with priority, which are summarized in Table 1.

Considering the high rates of gastrointestinal side effects of miltefosine and lack of regular food intake in endemic settings, the group noted the importance of investigating formulations with reduced gastrointestinal side effects. The absence of any side effects was also one of the key features noted in the PPC (Table 2). The development of simplified weight-band-based dosing tables for the proposed allometric dosing of miltefosine was also noted, with considerations around harmonizing weight bands with those used for other diseases that are common in VL high-burden settings.

Establishing specific exposure-response relationships for VL in relation to the target exposure for children was noted as a key priority to optimize dosing strategies for children. These studies, conducted only for miltefosine in specific settings, should be undertaken in all regions where VL is endemic to account for regional specificities and their effect on medicine PK.

## Conclusions

Due to limited financial incentives, few new drugs are being developed for NTDs. In particular, children with NTDs are often left behind in accessing the latest available innovations that are suitable for them. This is particularly concerning given that several NTDs, such as VL, have a high burden in this vulnerable population. Research conducted in 2022 showed that, overall, less than half of WHO-recommended medicines for NTDs are approved for children, highlighting the urgent need to increase research activity for NTDs for children, giving priority to diseases that represent a significant burden and lack adequate treatment options (25).

The PADO for VL was the first ever exercise conducted to determine a clear set of priorities for children with VL by building consensus from various actors in the VL community. This will help researchers, developers and other stakeholders to focus efforts and mobilize funding around the identified priorities to ensure that children with VL have access to the latest available innovations that are suitable for them. The addition of a paediatric formulation of miltefosine to the WHO Prequalification expression of interest will promote generic production of this key formulation.

The PADO priorities will be monitored and, if needed, reviewed and revised in the future to guarantee alignment of the priorities to the latest evidence from ongoing and planned studies and development programmes.

This work represents a critical first step in prioritizing paediatric formulations for visceral leishmaniasis, contributing to a broader, coordinated effort to accelerate the development and equitable introduction of optimal treatments for children in resource-limited settings.

## Data Availability

The original contributions presented in the study are included in the article/Supplementary Material, further inquiries can be directed to the corresponding author.
