# Supplementary material for: Optimization and prioritization of paediatric drugs for visceral leishmaniasis
Source: Front Pediatr. 2025 Sep 4;13:1635252. doi: 10.3389/fped.2025.1635252 (PMC12443688; doi:10.3389/fped.2025.1635252)
Supplement: Supplementary file 1 [file Table1.docx]

**Optimization and prioritization of paediatric drugs for visceral leishmaniasis**

Tiziana Masini, Ana Nilce Silveira Maia-Elkhoury, Dinesh Mondal, Piero Olliaro, Khechar N. Paudel, Martina Penazzato, Samantha Yuri Oshiro Branco Valadas, Aya Yajima, Abate Beshah, Supriya Warusavithana, Saurabh Jain

Supplementary information

**Table S1.** Summary of visceral leishmaniasis burden and treatment options by World Health Organization region

| **WHO region** | **Main observations** |
| --- | --- |
| **Region of the Americas** | - In the region, there are 13 endemic countries, with an average of 2,990 cases per year (and a decreased trend in cases in 2023, with 1,461 cases of visceral leishmaniasis (VL)); the majority of cases (91%) occur in Brazil. - VL-HIV co-infection is an increasing problem, with 19% cases in 2023. This requires careful interpretation as the total number of VL cases have gone down, while the percentage of coinfection has gone up. - Case fatality rate in 2023 was 8%. Majority of deaths in HIV-VL cases. - A high VL burden and mortality rate are reported in children aged 5 years and below (21,7% and 16.6%, respectively). (Maia-Elkhoury ANS, Sierra Romero GA, Valadas SYOB, E et al. Premature deaths by visceral leishmaniasis in Brazil investigated through a cohort study: a challenging opportunity? *PLoS Negl Trop Dis*. 2019; **13**:e0007841) - Procurement of medicines through the Pan American Health Organization Strategic Fund supports countries in the region to procure VL drugs at negotiated prices irrespective of the disease burden. - Liposomal amphotericin B is recommended in paediatric and adult non-immunocompromised patients, but it is available only in 50% of the endemic countries and where available, there is low use given low confidence by clinicians. |
| **South-East Asia Region** | - 6 countries are endemic for visceral leishmaniasis. - In 2013-2023, around 61,081 cases have been treated for VL in the region. Since 2002, miltefosine (capsules) have been registered and used to treat VL (28 days of oral therapy) until 2013-14 when it was replaced with liposomal amphotericin B as the first line treatment. Miltefosine is also under use for the long-term treatment (12 weeks or oral therapy) of post-kala-azar dermal leishmaniasis (PKDL). (WHO guideline development process has recommended replacing the 12-week treatment due to the lack of safety of this regimen. (WHO guideline is under print) - The majority of people with VL aged between 0-15 years have been treated with liposomal amphotericin B, or miltefosine. Most children with VL are malnourished and with secondary infections, highlighting the importance of ensuring routine nutritional assessment, management of anaemia and infections. - In Nepal, more than 30% of the total VL cases in 2021 were in people aged 14 years or below. - Acceptability of VL treatments is good for children and caregivers, but administration in children is challenging (in particular, administration of IV infusions is challenging and issues with swallowing of miltefosine capsules and long duration of treatment were noted during the meeting). - Recently, there are reports of around 70 cases of ocular adverse events in PKDL patients administered with miltefosine. As per the data available around 28% of these eye complications among PKDL cases are in the age group up to 17 years. |
| **African Region** | - 15 countries are endemic for visceral leishmaniasis. - Eastern African countries (Eritrea, Ethiopia, Kenya, South Sudan, and Uganda) currently account for the highest VL burden worldwide, with the maximum burden being in Kenya, Ethiopia and South Sudan in the World Health Organization (WHO) region of Africa. - In Ethiopia, the majority of people with VL are above 15 years of age, while in Kenya, South Sudan and Uganda, the majority of people with VL are below 15 years of age. - In all countries, there is an increased trend of number of cases observed between 2018 and 2022, including increasing trends in younger children. One of the reasons is improved surveillance, uninterrupted supplies of rapid diagnostic tests and drugs, or focal outbreaks etc. - The first-line treatment regimen is the two injections of pentavalent antimonials plus paromomycin for a minimum of 17 days. The intravenous infusion of liposomal amphotericin B is reserved for special conditions e.g., pregnancy, young children, very sick patients, malnutrition, VL-HIV coinfection, relapse from the first-line therapy, or in patients who are not eligible for the first line therapy etc. - The majority of children with VL are malnourished (especially those below 5 years of age), so administering injections is difficult because of poor muscle mass, and acceptability by children is low because of painful injections and the risk of thrombosis of veins. - Comorbidities are frequently encountered, such as pneumonia and other respiratory infections and intestinal infections. - While diagnostic testing for VL and VL medicines are free of charge, medication costs for opportunistic infections and bed fee and investigations other than VL testing in some facilities are covered by families. - Médecins Sans Frontières (MSF) has been involved in the management of VL in South Sudan since 1993. Between 2014-2022, 61.4% of people with VL treated by MSF was 0-14 years, with 80% of them experiencing acute malnutrition, with malnutrition affecting especially children below 2 years of age. Anaemia is also very common among children admitted for VL treatment, with clear improvement observed after treatment. (Kämink SS, Collin SM, Harrison T, et al. A clinical severity scoring system for visceral leishmaniasis in immunocompetent patients in South Sudan. *PLoS Negl Trop Dis* 2017; **11**:e0005921) - All antileishmanial drugs and diagnostics are supplied through external support |
| **Eastern Mediterranean Region (Djibouti, Somalia and Sudan)** | - 18 countries are endemic for visceral leishmaniasis. - Since 2019, Sudan reports the highest VL burden globally. Other high burden countries are Somalia, Yemen, Iraq (reporting more than 100 cases annually). - Children and young adolescents aged 0 to 14 years represent approximately 70% of the VL burden in Sudan, with a mean age of people with VL in the endemic areas being 8.6 years and malnutrition being one of the major risk factors (Zijlstra EE, el-Hassan AM. Leishmaniasis in Sudan. 3. Visceral leishmaniasis. *Trans Royal R Soc Trop Med Hyg.* 2001;**95**: S27–58) - Post-kala-azar dermal leishmaniasis used to be of high incidence in East Africa (>50%) and especially in Sudan, but incidence is reducing lately (12%). An ongoing large cohort study is in preparation in Sudan and will provide more information about the burden of PKDL. - While children prefer oral therapy, parents prefer children to receive parenteral therapies, with the belief that they are more effective. - Two treatments are available as the first line regimen in Sudan, namely paromomycin and sodium stibogluconate. The second line treatment, liposomal amphotericin B is not suitable for endemic areas where storage facilities and cold chain are suboptimal, so the drug is reserved to tertiary hospitals in Sudan or research centres and shortages are very often. - Price, availability and supply of VL medicines is an issue, with unsustainable supply resulting in unavailability due to last mile supply chain challenges. - All antileishmanial drugs and diagnostics are supplied through external support. |
| **Western Pacific Region** | - Only China is endemic for visceral leishmaniasis in the Western Pacific Region. China reports around 200 new VL cases annually. - The epidemiology of visceral leishmaniasis in China is diverse and complex, but 3 nosogeographical entities can be identified, namely:   - i) anthroponotic form in eastern plains   - ii) zoo-anthroponotic form in mountainous and hilly regions of central and north-west China, (the causative species is *L. infantum)* and,   - iii) anthroponotic form in desert regions of eastern China (the causative species is *L. donovani)* |
